# Idiopathic toe-walking in children, adolescents and young adults: a matter of local or generalised stiffness?

**DOI:** 10.1186/1471-2474-12-61

**Published:** 2011-03-21

**Authors:** Raoul Engelbert, Jan Willem Gorter, Cuno Uiterwaal, Elise van de Putte, Paul Helders

**Affiliations:** 1 Education of Physical Therapy, Amsterdam School of Health Professions, University of Applied Sciences (Hogeschool van Amsterdam), Amsterdam, The Netherlands; 2 Department of Rehabilitation, Academic Medical Center, Amsterdam, The Netherlands; 3 CanChild, Centre for Childhood Disability Research, Department of Pediatrics, McMaster University, Hamilton, Canada; 4 Julius Centre for Health Sciences and Primary Care, University Hospital for Children and Youth 'Wilhelmina Children's Hospital', University Medical Center Utrecht, Utrecht, the Netherlands; 5 Department of Pediatrics, Wilhelmina Children's Hospital, University Medical Center Utrecht, the Netherlands; 6 Department of Pediatric Physical Therapy and Pediatric Exercise Physiology, Wilhelmina Children's Hospital, University Medical Center Utrecht, the Netherlands

## Abstract

**Background:**

Idiopathic Toe Walking (ITW) is present in children older than 3 years of age still walking on their toes without signs of neurological, orthopaedic or psychiatric diseases. ITW has been estimated to occur in 7% to 24% of the childhood population. To study associations between Idiopathic Toe Walking (ITW) and decrease in range of joint motion of the ankle joint. To study associations between ITW (with stiff ankles) and stiffness in other joints, muscle strength and bone density.

**Methods:**

In a cross-sectional study, 362 healthy children, adolescents and young adults (mean age (sd): 14.2 (3.9) years) participated. Range of joint motion (ROM), muscle strength, anthropometrics sport activities and bone density were measured.

**Results:**

A prevalence of 12% of ITW was found. Nine percent had ITW and severely restricted ROM of the ankle joint. Children with ITW had three times higher chance of severe ROM restriction of the ankle joint. Participants with ITW and stiff ankle joints had a decreased ROM in other joints, whereas bone density and muscle strength were comparable.

**Conclusion:**

ITW and a decrease in ankle joint ROM might be due to local stiffness. Differential etiological diagnosis should be considered.

## Background

Toe-walking is the inability to generate a heel strike during the initial contact phase of the gait cycle, the absence of full foot contact during the entire standing phase and is a common pattern seen in healthy developing children less than 3 years old [1]. Idiopathic Toe Walking (ITW) is present in children older than 3 years of age still walking on their toes without signs of neurological, orthopaedic or psychiatric diseases [2]. ITW has been estimated to occur in 7% to 24% of the childhood population [3].

Toe-walking without a decrease in range of joint motion of the ankle joint is reported to be present in children with autism, in children with communication (language) disorders and learning disabilities, as well as in children with fine motor, visuomotor- and gross motor delays [4,5].

No consensus is found in the literature between the presence of ITW and a decrease in local range of motion (ROM) (ankle joint). Shulman et al. reported that over 50% of the children who walked on their toes had normal or only mildly limited dorsal extension of the ankle joint^, ^[5] whereas Sobel et al. reported a severe decrease in range of joint motion (mean: -5.2 degrees) in 46% [6]. Westberry et al. analyzed walking patterns with quantitative gait analysis in 51 children with ITW and asked these children to attempt a normal heel-toe gait. Nine of 51 (17%) were able to normalize both stance and swing variables whereas 35 of 51 (70%) were able to normalize some but not all stance and swing variables. Mean (sd) dorsal extension of the ankle joint with full extension in the knee joint was -1.8 (8.5) degrees [7].

ITW with decreased range of joint motion in the ankle joint was present in a majority of children with musculoskeletal complaints and stiffness of other joints defined as symptomatic generalised joint hypomobility. Generalised joint stiffness which may lead to musculoskeletal complaints was possibly caused by an increased amount of stiffness of the connective tissue, as illustrated in skin biopsies (increased collagen and cross-linking contents) [8].

In present study, we studied the association between a positive history or present occurrence of ITW and a decrease in ROM of the ankle joint at assessment.

Secondly, we studied the association between ITW (with decreased ROM of the ankle joint) and decreased ROM in other joints.

Thirdly, we studied muscle strength and qualitative measurements of bone in order to study if ITW is a local or generalized pattern and thereby provide more insight in the (patho)physiology.

## Methods

### Study design and inclusion/exclusion criteria

In a cross-sectional study, 362 children, adolescents and young adults (mean age (sd): 14.2 (3.9) years, range: 8.0 - 19.9; 236 girls, 126 boys) participated. One hundred and seventeen out of 362 participants were recruited from from primary schools (age 8 - 11 years; 65 girls, 52 boys), whereas 167 out of 362 participants (age 12 - 17 years; 101 girls, 66 boys) were recruited from a secondary school in the city Zeist, the Netherlands. Another 78 participants were recruited from the University of Applied Sciences, Education of Physiotherapy, in Utrecht, the Netherlands (age 18 - 20 years; 70 girls, 8 boys). All participants served also in a age-matched reference group for research involving children with symptomatic generalized joint hypermobility and joint hypomobility. All participants were healthy without pathophysiological conditions involving the musculo-skeletal system [8,9].

We defined ITW as present when toe walking without heel strike was present for at least three months with the absence of past or present signs of any rheumatic, neurologic, skeletal, metabolic, or collagen disease.

The Medical Ethics Committee of the Wilhelmina Children's Hospital (University Medical Center Utrecht) approved this study, while informed consent was obtained from all parents (when indicated due to age) and participants.

### Measurements

We asked about ITW by questionnaire and encouraged participants to ask their parents if toe walking was present for a period of more than 3 months after three years of age. We also asked if any medical care was provided for this reason from birth onwards.

The active range of joint motion of the shoulder (anteflexion), elbow (flexion and extension), wrist (palmar- and dorsal extension), hip (flexion and extension), knee (flexion and extension) and ankle (plantar and dorsal extension) was measured bilaterally to the nearest 2 degrees with a standard 2-legged 360-degree goniometer, using the "anatomical landmark" method [10]. In the upper extremity, shoulder ROM was assessed in sitting position, whereas flexion and extension of the elbow were assessed with 90 degrees flexion in the shoulder joint (sitting position). Palmar- and dorsal extension in the wrist joint were assessed with 90 degrees flexion in the elbow joint (sitting position).

The ROM of the lower extremity was assessed when the participant was positioned in supine.

A severe decrease in active dorsal extension of the ankle joint, measured in supine position with full extension of the knee joint, was defined when active dorsal extension, was ≤ 0 degrees. Since the position of the knee joint affects the amount of dorsal extension of the ankle joint, 0 - degrees extension is the knee joint is indicated.

Before start of the study, inter- and intrarater reliability was measured. Two assessors had to measure 25 joint excursions in 12 participants. All measurements were performed by the same trained physical therapists under supervision of the first author.

The mean difference between two measurements of active ROM within one assessor (intra-rater) was 2.4 degrees (sd: 4.6). The mean difference between two measurements of active ROM within two assessors (inter-rater) was 5.0 degrees (sd: 4.7). These data were gathered in joints with large excursions (e.g. shoulder joint: flexion) as well as in joints with small excursions (e.g. elbow extension). The results of the reliability study indicate that 95% (= 2 standard deviations) of the difference between all measurements was less than 9.4 degrees which we found acceptable for measuring range of joint motion. When analyzing intrarater reliability of the ankle joint only, the mean (sd) difference was 1.9 (2.5) degrees indicating that 95% (= 2 standard deviations) was less than 5.0 degrees. The left and right-sided values of the ROM were averaged. We analysed range of joint motion of all excursions bilaterally. Only in the extension of the elbow, dorsal extension of the wrist and ankle, a significant difference existed (mean (sd) ROM elbow: left - right: 5.0 (6.1) - 4.4 (6.0); p-value: 0.004, mean (sd) ROM wrist: left - right: 75.8 (9.4) - 75.1 (9.1); p-value: 0.02, mean (sd) ROM ankle: left - right: 7.8 (6.2) - 8.3 (6.3); p-value: 0.01 respectively. To provide an indication of ROM in all joints, total range of joint motion was a summation of all measurements without that of the ankle joint. Measurements were performed by asking the participants to actively stretch or bend the joint maximally without interference by the investigator, and without support of the ipsilateral muscles by use of contralateral limbs.

Maximum voluntary isometric muscle strength (expressed in Newtons) of the shoulder abductors, hip flexors, ankle dorsal flexors, and grip strength was measured bilaterally. The 'break' technique was used to test muscle strength. This technique is defined as 'the examiner applying force to a participant's limb until the child's capacity to hold is exceeded and the limb gives way". This methode has been shown to be reliable [11]. Measurements were performed with a hand held dynamometer (CIT Technics, Groningen, the Netherlands). Verbal encouragement was given during the attempts to achieve maximal effort. The highest value of three attempts was registered. The left and right-sided values of the muscle groups were averaged. Total muscle strength was calculated by adding up the separate scores.

Weight (in kilograms) was measured using an electronic weight scale. Height (in meters) was measured using a wall-assembled stadiometer. Body Mass Index (BMI) was calculated with the formula weight/height [12].

We asked the participants to estimate the mean amount of hours spent on sport activities during last month, we asked about the occurrence of musculoskeletal complaints after sport activities and the presence of injuries.

Quantitative ultrasound (QUS) measurements of the right os calcis were performed, according to the protocol of the manufacturer. A non-invasive method for assessing bone quantity and bone stiffness measurements was used (Sahara ultrasound device, Hologic QDR 4500, Hologic Inc, Waltham, MA) and broad band ultrasound attenuation (BUA, dB/MHz) and speed of sound (SOS, m/s) were measured [13,14]. Acoustic phantoms, provided by the manufacturer, were scanned daily and showed no drift over the time span of the study.

### Statistical analysis

Data were analyzed using SPSS 16.0 (SPSS Inc, Chicago, Ill).

Normally distributed data are described with descriptive statistics (mean, minimum, maximum, range and standard deviation scores).

The Odds ratio was calculated for associations between dichotomous variables (*ITW *(yes-no) and restricted joint motion of the ankle joint as outcome (yes-no) with a 95% confidence interval (95% CI).

We analysed with a paired T test range of joint motion of all excursions bilaterally.

Regression analysis (univariate) was performed to compare clinical characteristics in participants with ITW and restricted ROM of the ankle joint versus controls. Results are expressed as a regression coefficient with a 95% confidence interval (CI). In multivariate regression analysis adjustments were made for confounding variables as age, gender, body height and weight. For all tests p-values <0.05 (two-tailed) were considered statistically significant.

**Figure 1:**
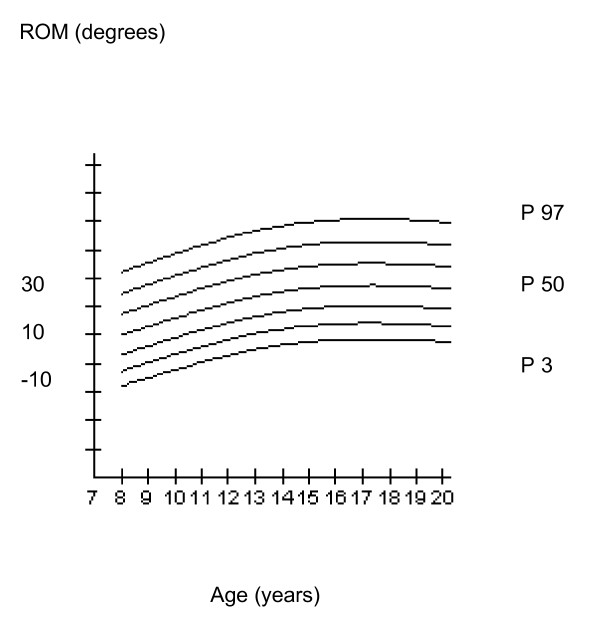
**Range of joint motion in the ankle joint, between 8 and 20 years of age**. P50: Fiftieth percentile. P3: Third percentile. P97: Ninety-seventh percentile.

Smoothed centile curves were computed (Figure 1) by using the LMS method (LMS-chartmaker Pro version 2.0, Medical research Council, UK).

## Results

### Sample description

Three hundred forty eight of 362 participants provided detailed information about the presence of ITW, so 14 participants were excluded.

### Patient outcome

Thirty-one of 348 (9%) participants reported ITW and had a decreased ROM of the ankle joint (9 of 122 boys (7%) and 22 of 226 girls (10%). In 14 of 348 participants (4%), decreased ROM of the ankle joint was found without a history of ITW. In 9 of 348 (3%) ITW was present without a decreased ROM of the ankle joint. Two-hundred ninety three of 348 (84.4%) did not report ITW and no decrease in ROM of the ankle joint was present. Participants with ITW had a 3.2 times greater chance of having a decrease in ROM of the ankle joint (Odds Ratio: 3.2, 95% CI: 2.1 - 5.0).

Data from participants with ITW and decreased ROM of the ankle joint (n = 31) compared to participants without ITW and without decrease in ROM is are presented in Table 1. Mean (sd;range) of dorsal extension in this group was -0.4 degrees (1.3; 0 - -5) whereas in the group without ITW and without decrease in ROM of the ankle joint was 9.2 (5.2;0.1-22.5) degrees (Table 1.)

**Table 1:** Univariate analysis between ITW and restricted range of joint motion in the ankle joint versus controls.

	ITW and restricted range of joint motion in the ankle joint (n = 31)	Controls (n = 317)	Mean difference (95% CI)
	Mean (sd)	Mean (sd)	
Age (years)	15.2 (2.0)	14.0 (4.0)	-1.2 (-2.6, 0.3)
Body height (cm)	170.9 (10.5)	160.6 (18.6)	**-10.2 (-16.9, -3.6)**
Body weight (kg)	59.3 (10.7)	51.6 (17.2)	**-7.8 (-13.9, -1.6)**
			
Hours sport activities/week	3.1 (0.7)	2.9 (2.1)	-0.2 (-1.0, 0.5)
Total range of joint motion (degrees)	1403.4 (62.9)	1428.7 (57.9)	**25.35 (3.7, 47.0)**
Range of joint motion ankle joint dorsal extension (mean, sd; range)	- 0.4 (1.3; 0 - -5)	9.2 (5.2;0.1-22.5)	**-9.2 (-11.1, -7.2)**
Total muscle strength (N.)	1326.1 (313.2)	1140 (372.8)	**-185.5 (-323.4,-47.5)**
Quantitative bone ultrasound BUA (dB/MHz)	68.9 (13.5)	65.5 (16.6)	-3.4 (-9.5, 2.6)
Quantitative bone ultrasound SOS (m/s)	1556.5 (25.2)	1567.7 (29.0)	**11.1 (0.5, 21.8)**

Values are means (sd); BUA: broadband ultrasound attenuation, SOS: speed of sound. Total range of joint motion was a summation of all measurements without the ankle joint. Statistically significant associations are given in bold face.

Children with ITW and restricted ROM of the ankle joint were significantly taller and weighted more than the control group. Total range of joint motion and quantitative bone density were significantly decreased, whereas total muscle strength was significantly increased. After adjustment for age, gender, body height and weight, only a significant decrease in total range of joint motion remained (regression coefficient (95% CI): 21.9 (2.1 - 41.7) (Table 2)

**Table 2:** Multivariate analysis between ITW and restricted range of joint motion in the ankle joint versus controls, adjusted for confounders.

	ITW and restricted range of joint motion in the ankle joint (n = 31)	Controls (n = 317)	Mean difference (95% CI)	Adjusted mean difference (95% CI)
	Mean (sd)	Mean (sd)		
Total range of joint motion (degrees)	1403.4 (62.9)	1428.7 (57.9)	**25.35 (3.7, 47.0)**	**21.9 (2.1, 41.7)**
Total muscle strength (N.)	1326.1 (313.2)	1140 (372.8)	**-185.5 (-323.4,-47.5)**	-19.5 (-86.7, 47.6)
Quantitative bone ultrasound BUA (dB/MHz)	68.9 (13.5)	65.5 (16.6)	-3.4 (-9.5, 2.6)	
Quantitative bone ultrasound SOS (m/s)	1556.5 (25.2)	1567.7 (29.0)	**11.1 (0.5, 21.8)**	7.9 (-18.4, 2.6)

Values are means (sd); BUA: broadband ultrasound attenuation, SOS: speed of sound. Total range of joint motion was a summation of all measurements without the ankle joint. Statistically significant associations are given in bold face.

Adjustments were made for age, gender, body weight and height

Children who were idiopathic toe-walkers with a decrease in ROM of the ankle joint were as active as controls as reported by the hours spent on sport activities. The cut-off point for severe decrease in ROM of the ankle joint is arbitrary. In the present study we chose 0 - degrees dorsal extension of the ankle joint as a severe decrease in ROM. No significant differences in gender were found for the ROM of the ankle joint. When participants grew older, ROM tended to increase (Figure 1).

**Figure 2:**
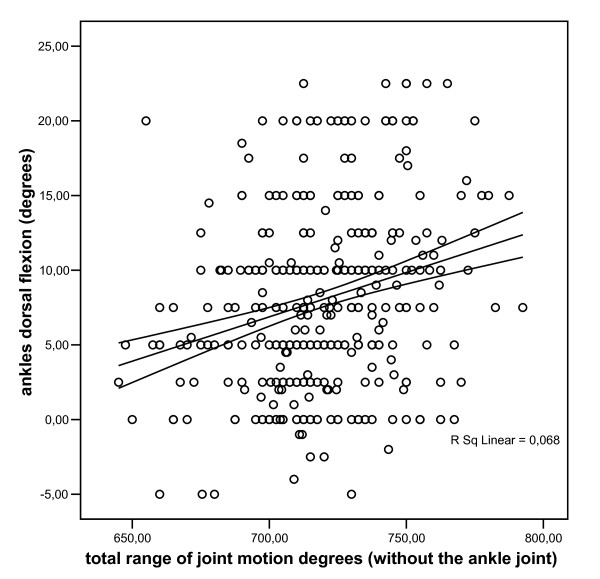
**The association between the amount of dorsal flexion of the ankle joint with the total range of joint motion of the joints of the right upper and lower extremities (without the range of joint motion of the ankle joint)**.

The association between the amount of dorsal extension of the ankle joint with the total ROM of all joints without the ankle joint is illustrated in Figure 2.

Regression analysis revealed after adjustment for gender, age, body height and weight that the amount of dorsal extension of the ankle joint was positively associated with the total ROM of the other joints (regression coefficient (95% CI): 2.8 degrees (1.8 - 3.9).

Total range of joint motion was significantly negatively associated with body weight (regression coefficient (95% CI): -0.5; (-0.9 - -0.2) and gender (regression coefficient;95% CI: -41 (28.7 - 52.9). These observations did not change after multivariate analysis.

## Discussion

In a general population of children, adolescents and young adults in the Netherlands we found a prevalence of 12% for ITW. Nine percent had a history of ITW and severely restricted ROM of the ankle joint. Participants with ITW had a three times higher chance of a severe restriction in their active dorsal extension of the ankle joint. After adjustment for confounders, participants with ITW and decreased ROM of the ankle joints had a significantly decreased range of joint motion in other joints, whereas quantitative ultrasound measurements and muscle strength were comparable.

Several intrinsic limitations of this study might have influenced the findings and should be taken into account. The nature of the present study is cross-sectional and information on the presence of ITW in the past was obtained retrospectively from the participants. The children and adolescents might not have recalled the presence of ITW leading to an underestimation of the prevalence, although they were encouraged to ask their parents. However, we feel reassured that the prevalence of ITW in our study is comparable with data in the literature where ITW has been estimated to occur in 7% to 24% of the childhood population [3].

Another intrinsic limitation was that we asked the participants to estimate the mean amount of hours spent on sport activities during last month. In future studies physical activity could be quantified by using a physical activity questionnaire.

The "diagnosis" ITW was based on retrospective data gathered by a questionnaire. Data form medical records (eg. surgery, physiotherapy treatment, period and time span of ITW) were lacking. Although these observations were not the primary goal of our study, these data might be very important to look into the different history of treatment and outcome.

The amount of stiffness was measured as a result from ROM. In future studies, it would have been interesting to use a dynamometer to quantify precisely the amount of stiffness.

Localized stiffness as a possible cause of ITW fits 9% of our study sample. However, 6.3% of your sample did not fit this theory (either ITW without stiffness, or stiffness without ITW). A comparatively large group did not fit in our theory.

ITW without stiffness was assessed in 3%. This might be explained by the fact that toe walking is often observed in children as a transient developmental expression [15].

Stiff joints without the presence of ITW might be explained that although children have a severe decrease in the ROM of their ankle joint, this is not always translated into toe walking, but more in compensatory valgus of the hindfoot with an exotorsion of the upper and lower leg. Compensatory strategies are frequently observed, especially in older children in which weight increases. Therefore the compensation in the walking pattern might not be recognised and reported as ITW.

Toe walking is often observed in children as a transient developmental expression and might eventually resolve spontaneously in the majority of children [15]. On the other hand, no data are reported on ROM of the ankle joint in this study. So, despite a spontaneous resolution of toe-walking, a decrease in ROM of the ankle joint might remain present.

The study populations were examined as a control group for children with symptomatic generalised hypermobility and hypomobility. Therefore only a history of toe-walking was available. As a consequence of the design of the study, no observational gait analysis could be performed regarding their current walking pattern.

In our study, we assessed active ROM because we are interested in the functional ability of the children's joint status. Passive ROM provides information of the maximum ROM of a joint but the standardization of the extended external force to reach passive ROM is discutable.

The cut-off point for defining decrease in range of joint motion is arbitrary. We defined a severe decrease in ROM of the ankle joint when the ankle could not actively dorsally extend more than 0 degrees with full extension of the knee joint. The position of the knee joint could have influenced our findings. However, no slight lack of full extension or slight hyperextension was observed.

We chose the above mentioned cut-off point because interpretation in measuring the ankle joint ROM by goniometry is easy to perform. Moreover, the 0 degree position of the ankle-foot was clearly defined with the foot is positioned perpendicular to the table. This method is also reported in other studies [7,16].

In the literature, the amount of toe-walking is primarily focussing on gait analysis and kinematics [16]. Little attention is focussed on ROM of the ankle joint. Hemo et al. reported on fifteen children who were diagnosed with idiopathic toe walking that cannot be corrected by nonoperative treatment. They were assessed by clinical examination and computer-based gait analysis preoperatively and approximately one year after Achilles tendon lengthening [17]. Passive dorsiflexion improved from a mean plantarflexion contracture of 8 degrees to dorsiflexion of 12 degrees after surgery. Ankle kinematics normalized, with mean ankle dorsiflexion in stance improving from -8 to 12 degrees and maximum swing phase dorsiflexion improving from -20 to 2 degrees.

The combination of ITW and decreased ROM of the ankle joint has been presented by Alvarez et al. who studied gait analyses in 133 children with toe-walking (mean age (yrs): 9, range: 4-16) and classified toe-walking in three severity groups, based on range of joint motion, sagittal joint powers, knee kinematics and EMG data [16]. Ninety of 266 (34%) feet had type 3 ITW (severe). In this group, the mean (sd) ROM of the ankle joint was -1.6 (10.5) degrees.

Reference data on ankle ROM in children are scarce. Engelbert et al. reported in a group of 284 children a mean (sd, range) active ROM of 9 degrees (6;-5 - 25 degrees) [9]. We suggest that a decrease in active ROM between -1 and -2 sd from normal (translated in degrees between 3 and -3 degrees) should be considered as severe decrease in ROM, eventually resulting in toe walking (active dorsal extension in the ankle joint of 0 degrees (plantigrade position in supine lying with extended knee). When in our present study a severe decrease in ROM should be defined as ROM less than -2 sd (-3 degrees), only 7 participants should be included.

An advantage of our study was that we performed an intrarater and interrater reliability study concerning range of joint motion. We reported a mean difference between two measurements of 2.4 degrees (sd: 4.6) respectively 5.0 (sd 4.7) indicating that 95% (=2 standard deviations) was less than 9.2, respectively 9.4 degrees. This applies for all joints, also joints with a large range of possible joint motion, e.g. the shoulder, hip and knee joint. When analyzing reliability only of the ankle joint, the mean (sd) difference was 1.9 (2.5) degrees indicating that 95% (=2 standard deviations) was less than 5.0 degrees.

In this study, participants with ITW and decreased ROM of the ankle joint were as active as controls as reported by the same amount of hours spend on sport activities. Our study population did not report the occurrence of pain during sport activities. This is in contrast with the study by Engelbert et al. They described toe-walking with a decrease in ROM of the ankle joints in combination with musculoskeletal complaints (pain during sport activities). The authors also reported a decrease in ROM in other joints who were possibly due to increased collagen and cross-linking contents in skin biopsies [8].

In that study, the decrease in joint mobility in other than the ankle joint was more pronounced.

Children with ITW and decreased ROM of the ankle joint had a decrease in total ROM between 2 and 42 degrees (95% CI) in other joints than the ankle joint. Although significant, the clinical relevance is probably limited (22 from 1400 degrees = 2%).

Since we found no differences in bone quantitative measurements, muscle strength and clinically relevant differences in total ROM we think that ITW and decreased ROM of the ankle joints are rather a local stiffness than a local manifestation of a systemic problem. The findings of the study may have implications for treatment.

The amount of total ROM is gender related. In our study, total ROM in female is increased compared to male. In general, ROM decreases when growing older. Although the design of the study was cross-sectional, we observed in older participants a decrease in ROM in the proximal joints (shoulder- and hip joint) whereas in the distal joints ROM remained the same or slightly increased (data not illustrated).

In cases where decreased range of ankle-foot joint motion in ITW is present, treatment strategies to improve local ROM are not always successful. Stretching by a physiotherapist and casting protocols alone have not been shown to be successful with incomplete correction and high recurrence rates [18-20]. Also surgery can have improved outcomes in children with ITW, but not in all cases [21].

A group of young children where found to have good results with Botulinum Toxin A with the majority of children having a heel toe walking pattern 12 months following the injections [22]. This approach is in line with contemporary thinking about passive and active stretching of muscles with and without underlying stiffness of one or more joints [23,24].

In cross- sectional studies on ITW, we do advise to use disorder specific instruments. Recently, Williams et al developed the Toe Walking Tool, a valid and reliable method of assessing children who present to the general allied health clinician with toe walking. This tool can assist with the decision of when to refer a child for further specialist investigation of their toe walking [25,26].

Differential diagnostics is indicated to study the aetiology of ITW. Different diagnosis related to ITW in the literature are presented in Table 3[2,5,18,26-29].

**Table 3:** Differential diagnostics in ITW

**Differential diagnostics where ITW is present as symptom or marker **[2,5,18,26-29]
**Based on central nervous system disorders**

Cerebral palsy

Lesion of the tractus corticospinalis

Hyperkinesia disorders

Transient dystonia (unilateral ITW)

**Based on peripheral neurological disorders (spinal aberrations)**

Congenital/acquired spinal cord lesion

Diastematomyelia

Spinal tumour

Sensory processing disorders

**Based on peripheral neuromuscular disorders**

Peroneal muscular atrophy

Muscular dystrophy

Neuropathy

**Based on musculoskeletal disorders**

(Congenital) contracture of the achilles tendon or calf muscles

Osseal disorders as congenital club foot

Leg length discrepancies (unilateral ITW)

Arthrogryposis

**Based on developmental and pervasive disorders**

Autism/Autism Spectrum Disorder

Pervasive developmental disorder

Language, learning and developmental disorders

## Conclusion

In a general population of children, adolescents and young adults in the Netherlands. we found a prevalence of 12% for ITW. Nine percent had a history of ITW and a severe restricted range of joint motion of the ankle joint. Children who had been walking on their toes had an three times higher chance of a severe decrease in their active dorsal extension of the ankle joint in their adolescence compared to children without a history of idiopathic toe walking. Since we found no differences in bone qualitative measurements, muscle strength and clinical relevant differences in total range of joint motion we think that ITW and stiff ankle joints might be an expression of a local problem rather than generalized stiffness or systemic aetiology. Since the underlying aetiology of ITW is unknown, differential diagnostics regarding possible etiological factors should be considered.

## List of abbreviations

ITW: Idiopathic Toe Walking; ROM: Range of joint motion; BMI: Body Mass Index; BUA: Broad band ultrasound attenuation; SOS: Speed of sound; 95% CI: 95% confidence interval.

## Competing interests

The authors declare that they have no competing interests.

## Authors' contributions

RE, CU, EP and PH designed the study. RE, CU and EP performed data acquisition.

RE, CU, EP and JWG analysed and interpreted the data, RE wrote the first draft of the manuscript, which was reviewed by all authors. JWG revised the manuscript. All authors read and approved the final version.

## Pre-publication history

The pre-publication history for this paper can be accessed here:

http://www.biomedcentral.com/1471-2474/12/61/prepub
